# Measurement Properties of Questionnaires Assessing Complementary and Alternative Medicine Use in Pediatrics: A Systematic Review

**DOI:** 10.1371/journal.pone.0039611

**Published:** 2012-06-29

**Authors:** Karine Toupin April, David Moher, Jennifer Stinson, Ani Byrne, Meghan White, Heather Boon, Ciarán M. Duffy, Tamara Rader, Sunita Vohra, Peter Tugwell

**Affiliations:** 1 Department of Epidemiology and Community Medicine, Faculty of Medicine, University of Ottawa, Ottawa, Ontario, Canada; 2 Centre for Global Health, Institute of Population Health, University of Ottawa, Ottawa, Ontario, Canada; 3 Clinical Epidemiology Unit, Ottawa Hospital Research Institute, Ottawa, Hospital, Ottawa, Ontario, Canada; 4 Child Health Evaluative Sciences, The Hospital for Sick Children, Toronto, Ontario, Canada; 5 Lawrence S. Bloomberg Faculty of Nursing, University of Toronto, Toronto, Ontario, Canada; 6 Leslie Dan Faculty of Pharmacy, University of Toronto, Toronto, Ontario, Canada; 7 Department of Pediatrics, Children’s Hospital of Eastern Ontario, Ottawa, Ontario, Canada; 8 Department of Pediatrics, University of Ottawa, Ottawa, Ontario, Canada; 9 CARE Program, Department of Pediatrics, Faculty of Medicine and Dentistry, University of Alberta, Edmonton, Alberta, Canada; 10 School of Public Health, University of Alberta, Edmonton, Alberta, Canada; 11 Department of Medicine, University of Ottawa, Ottawa, Ontario, Canada; University of York, United Kingdom

## Abstract

**Objective:**

Complementary and alternative medicine (CAM) is commonly used by children, but estimates of that use vary widely partly due to the range of questionnaires used to assess CAM use. However, no studies have attempted to appraise measurement properties of these questionnaires. The aim of this systematic review was to critically appraise and summarize measurement properties of questionnaires of CAM use in pediatrics.

**Study design:**

A search strategy was implemented in major electronic databases in March 2011 and conference websites, scientific journals and experts were consulted. Studies were included if they mentioned a questionnaire assessing the prevalence of CAM use in pediatrics. Members of the team independently rated the methodological quality of the studies (using the COSMIN checklist) and measurement properties of the questionnaires (using the Terwee and Cohen criteria).

**Results:**

A total of 96 CAM questionnaires were found in 104 publications. The COSMIN checklist showed that no studies reported adequate methodological quality. The Terwee criteria showed that all included CAM questionnaires had indeterminate measurement properties. According to the Cohen score, none were considered to be a well-established assessment, two approached the level of a well-established assessment, seven were promising assessments and the remainder (n = 87) did not reach the score’s minimum standards.

**Conclusion:**

None of the identified CAM questionnaires have been thoroughly validated. This systematic review highlights the need for proper validation of CAM questionnaires in pediatrics, which may in turn lead to improved research and knowledge translation about CAM in clinical practice.

## Introduction

According to the National Center for Complementary and Alternative Medicine (NCCAM), complementary and alternative medicine (CAM), includes many types of therapies and products that are not considered to be part of conventional medicine [1]. NCCAM mentions four domains of complementary and alternative medicine: 1) natural products (e.g. vitamins, minerals, dietary supplements, probiotics); 2) mind-body medicine (e.g. meditation, yoga, acupuncture, deep-breathing exercises, guided imagery, tai chi); 3) manipulative and body-based practices (e.g. spinal manipulation, massage therapy); and 4) other CAM practices (e.g. movement therapies, traditional healers, energy therapies) [1]. Even though the NCCAM definition is widely known and used, there is no consensus on the definition of CAM [2] or the types of therapies or products that should be considered as such [3], which makes it difficult to collect data in a standardized manner in both research and clinical settings. Thus, it remains difficult to compare research from different studies and to ensure a thorough assessment of CAM use in clinical practice. Since the NCCAM definition is inclusive of many different types of therapies and products, we will use it in the present article.

There is an increasing interest in CAM in pediatrics [4], stressing the importance for health care providers to initiate a dialogue about CAM use in order to assess its impact on their lives and to provide the most up-to-date evidence-based advice on risks and benefits of these therapies. While research has been done in many pediatric populations, a recent systematic review of prevalence of CAM use in pediatric cancer patients has highlighted the lack of standardized methods and validated questionnaires to assess patients’ use and perceptions of CAM in this population [2]. Authors of that review urge the scientific community to develop standardized, valid and comprehensive CAM questionnaires. To date, no systematic review has assessed the extent of measurement properties of CAM questionnaires in pediatrics.

The objective of this systematic review was to critically appraise and summarize the research evidence on the measurement properties of questionnaires assessing the use of CAM in pediatrics. To achieve this goal, we adapted the research protocol for systematic reviews of measurement properties created by Terwee et al. which includes steps such as evaluating the methodological quality of the included studies and the content and measurement properties of the instruments used in those studies [5] (protocol shown in Appendix S1). The secondary objective of this review was to inform the development of a valid and reliable questionnaire assessing the use of CAM for discriminative and evaluative purposes in pediatric clinical and research settings (should one not already exist). We used the PRISMA statement to help guide the reporting of the systematic review (see completed PRISMA checklist in Appendix S2) [6].

## Methods

### Literature Search

An electronic search strategy (See Appendix S3) was developed by a librarian (TR) and refined using the peer review of electronic search strategies checklist [7]. The search strategy was implemented in the following electronic databases: MEDLINE (1950 to week 12 2011), Healthstar, Cochrane CENTRAL, AMED (1985 to 2011), PsycINFO, The Cochrane Collaboration’s Methodology Register, EMBASE (1980 to 2011) and the Health and Psychosocial Instruments database (1985 to the last week of March 2011). Conference websites, scientific journals and experts in the field of CAM were also consulted in order to identify relevant publications of CAM questionnaires.

### Eligibility Criteria

Questionnaires were included if they sought to assess the prevalence of a wide range of CAM use (more than one type or one sub-category of CAM), such as products and services provided by CAM practitioners, in pediatric patients (age 18 years and younger), and if they were reported in English or French language articles. All types of studies (e.g. psychometric studies, prevalence studies and clinical trials) were included if they reported using such a questionnaire. Grey literature, such as abstracts published in scientific journals or proceedings of conferences were excluded due to lack of information concerning questionnaires used. Results from our search were exported into a Reference Manager database.

### Identification of Studies

Two members of the team (KTA and AB or BD) independently screened identified titles, abstracts and key words for relevance, and assessed potentially relevant studies for inclusion according to our eligibility criteria. Full text articles of records meeting the screening criteria were obtained. Each reviewer then decided independently which articles met the eligibility criteria and should be included in the systematic review. Disagreements were resolved by discussion with a third member of the team (JS or DM). If some data from the studies were insufficient or missing, we attempted to obtain this information from the authors by personal communication (email) up to three times.

### Data Extraction Procedures

Data from included studies were independently extracted by three members of the team using the previously piloted data extraction form (KTA and MW or AB) and then entered into an Excel spread sheet. The extracted data included: characteristics of the studies (study design, objectives, geographical location, target population) and the questionnaires (purpose, child and/or proxy report, content, time to complete). The time to administer a tool was documented and rated as 1) short if it took less than 10 minutes, or 2) long if it took more than 10 minutes to complete [8].

The methodological quality of studies reporting each CAM questionnaire as well as the measurement properties of the CAM questionnaires themselves were assessed. When data were missing from a study, corresponding authors were e-mailed (two attempts) in an effort to obtain questionnaires and additional information. Authors were also e-mailed at the end of our systematic review in order to confirm results.

#### Assessment of the methodological quality of the studies

The methodological quality of the measurement property testing reported in the included studies was assessed using the COSMIN checklist (See Appendix S4) which is a validated tool that has been used to rate studies reporting measurement properties of health status measurement instruments [9]–[12]. The checklist includes the following properties: content, construct and criterion validity, test-retest, inter-rater, intra-rater reliability and measurement error, internal consistency and responsiveness (See definitions in Appendix S4) [11]. Interpretability and generalisability of studies can also be rated. The quality of each of the properties reported in a study is assessed by a series of items including design requirements and preferred statistical methods and can vary from excellent to poor depending on the information reported by the authors. A total score is determined by taking the lowest rating of the items for each measurement property.

#### Assessment of the measurement properties of the CAM questionnaires

We used the Terwee criteria [13] and the Cohen score [14] to assess measurement properties of the CAM questionnaires. Terwee et al. propose quality criteria for rating measurement properties [13]. Whereas the COSMIN checklist assesses the quality of the methods used in studies to validate a questionnaire, the Terwee criteria evaluate the results stemming from such studies. Validity, reliability, responsiveness, floor and ceiling effects and interpretability can be rated as positive, negative or indeterminate depending on the results of validation studies. Criterion validity was not applicable for CAM questionnaires since there is no gold standard to assess its use. Floor and ceiling effects were not applicable for items rated on a nominal scale such as CAM use, but could have been assessed for items which were rated on ordinal, interval and ratio scales. Interpretability was not applicable to CAM questionnaires since they did not provide a total score.

The Cohen criteria [14] (Shown in Appendix S5) seek to evaluate the degree of testing of measurement properties as well as the dissemination and description of the questionnaires. The Cohen criteria are divided into three levels depending on the number of publications describing the questionnaires, the extent to which questionnaires are described and their measurement properties.

### Synthesis

We completed a narrative summary of the included studies. Content analysis was used to study the content of the included articles. Descriptive statistics were used to describe the characteristics of the studies and questionnaires.

## Results

### Search Results

The electronic search provided a total of 476 records after eliminating duplicates. Additional consultation of conference websites, scientific journals and experts in the field of CAM yielded another 107 records. From these 583 unique potentially relevant records, 104 met the screening criteria. We included 104 articles in the systematic review which summarized 100 studies from which 96 CAM questionnaires were identified (see Figure S1 for a flow diagram of included studies and Appendix S6 for a list of references for the included studies). Among authors of included studies, 69 (69/96 = 71.9%) responded to our query by providing additional information on their questionnaires’ measurement properties. Some of these authors no longer had access to questionnaires and a total of 50 authors ultimately provided copies for this review. Although we did not receive questionnaires from all authors, six CAM questionnaires were displayed in their respective publications and one was found online. After completing our review, 51 (51/96 = 53.1%) authors confirmed our appraisal of their studies and questionnaires, nine of them gave new information that did not modify our assessment and one author gave us new information that modified our assessment of their questionnaire [15].

### Characteristics of the Studies

Included studies sought to assess children’s use of CAM (n = 100), perceptions regarding children’s use of CAM (n = 86) and attitudes towards CAM in general (n = 33). Most studies were cross-sectional surveys of CAM use (n = 98) and only two assessed CAM use longitudinally [16]; [17]. Only 58 studies provided a definition of what they considered to be CAM and definitions varied tremendously. The most common definition was the NCCAM definition (n = 18 studies) [1].

Studies were conducted in 19 countries, the most common being the United States (n = 35), United Kingdom (n = 15), Canada (n = 11), Australia (n = 8), Turkey (n = 6), Israel (n = 3), Ireland (n = 2), Germany (n = 2), Switzerland (n = 2) and New Zealand (n = 2). Three studies did not disclose precisely where they were conducted and three studies were conducted in multiple countries. Studies were conducted in general pediatrics (n = 24), in specific pediatric conditions (n = 63), such as cancer (n = 19), neurological (n = 16), respiratory (n = 11), gastrointestinal [5], rheumatologic (n = 4), dermatologic (n = 3), haematological (n = 2), metabolic (n = 2) and otolaryngological conditions (n = 1), as well as in both general pediatrics and specific diseases (n = 5). Other studies also assessed CAM use in children undergoing surgical procedures (n = 2), children with a range of diverse chronic diseases (n = 3) or acute illnesses (n = 2) as well as homeless youth (n = 1).

### Characteristics of the CAM Questionnaires

All included CAM questionnaires sought to discriminate between users and non-users of CAM (i.e. discriminative purpose) and none were reported to be designed to assess changes in CAM use over time (evaluative purpose). Thirty nine percent (n = 37) of the questionnaires assessed disease-specific CAM use while 42% (n = 40) assessed the use of CAM in a general pediatric population without referring to a specific disease, and one questionnaire assessed both current disease-related use as well as use for other reasons. Other questionnaires were not described in enough detail to determine whether they were generic or disease-specific (n = 18, 19%). Most questionnaires were targeted at parents, while 23% of questionnaires gathered children and adolescent self-reports and only two studies mentioned using a questionnaire specifically designed to gather children and adolescents self-report. Children completed questionnaires on their own beginning at 10 years of age while children as young as seven years helped their parents to answer the questions.

The content of the questionnaires varied considerably. The number of types of CAM listed in questionnaires ranges from 3 to 161, some questionnaires listing only broad categories of CAM (n = 7) and others presenting an exhaustive list of herbal products, vitamins and minerals. CAM types and categories were often not adequately reported by studies and their diversity made it difficult to synthesize the data. Questionnaires also included a wide range of domains and items linked to CAM use (see Figure S2). The most common domains and items were: reasons for CAM use (n = 61, 63.5%), helpfulness (n = 58, 60.4%), communication with health providers concerning CAM (n = 51, 53.1%), source of information about CAM (n = 48, 50%), CAM use by parents/family (n = 40, 41.7%) and costs of CAM (n = 38, 39.6%).

Concerning ease of use of the questionnaires, only ten were reported to have a completion time under 10 minutes and the others were reported to take up to 60 minutes to complete. A summary of the characteristics of questionnaires tested for measurement properties is shown in Table S1.

### Methodological Quality of the Studies

Using the COSMIN checklist assessment, none of the studies showed adequate methodological quality. Of the 11 questionnaires which were mentioned to have been tested for content validity [18]–[28], no studies were descriptive enough to reach the COSMIN criteria. Most of the publications (82%) describing those questionnaires did not mention whether items refer to and comprehensively represent relevant aspects of the construct to be measured. They also did not state whether items are relevant for the population surveyed or for the purpose of the questionnaire. Two CAM questionnaires [20]; [21] had their content validity assessed by both patients and experts, which is an advantage [13]; [29], while the other nine had it assessed by either patients (n = 2) or experts (n = 7).

Three CAM questionnaires were assessed for construct validity [15]; [30]; [31] but none were adequately reported. Most authors did not provide information on missing items, a priori hypotheses regarding the direction and magnitude of correlations, comparator instrument, sample size and statistical analyses. Authors of one study reported a small sample size (n = 25 patients) and the use of Spearman correlation coefficients by means of personal communication [15].

Of the four questionnaires which were reported to have been assessed for test-retest reliability [15]; [30]; [32]; [33], inter-rater reliability [30] or internal consistency [15], none was described in enough detail to meet the COSMIN criteria. Most of the publications describing those questionnaires did not report how missing items were handled and how repeated measurements were conducted (mode of administration, sample size, statistical analyses and time interval for test-retest). Some questionnaires were also tested with long delays between first and second administration (4 to 6 weeks), different modes of administration between test and retest, small sample size and inadequate statistical analyses. One questionnaire [15] showed variable levels of test-retest agreement using intraclass correlation coefficients as well as internal consistency using Cronbach’s alpha (data provided by authors by means of a personal communication).

### Measurement Properties of CAM Questionnaires

When evaluating the quality of measurement properties for each of the 96 included CAM questionnaires using the Terwee criteria, none was rated as thoroughly valid. No questionnaire reported an assessment of floor and ceiling effects. Only 16 CAM questionnaires were assessed for one or more of the following properties: content validity, and test-retest and inter-rater reliability (See summary characteristics of the most promising questionnaires in Table S1).

Ratings for content validity were indeterminate since questionnaires were not reported to have used appropriate methods (e.g. purpose of the questionnaire, concepts to measure, involvement of the target population, methods of item selection and reduction). None of the included questionnaires described their precise purpose (whether they are discriminative, evaluative or predictive) of the questionnaires. Measured concepts were often listed but not in a thorough manner. Only two questionnaires [22]; [25] were reported to be comprehensive (i.e. including all relevant items) and have involved the target population of patients. One showed a high level of agreement between experts ratings for included items [22].

Construct validity was rated as indeterminate for the three questionnaires [15]; [30]; [31]. The domain scores of one questionnaire were compared with measures of parental discipline and spiritual practice/beliefs, which yielded Spearman’s correlation coefficients ranging from −0.52 (p<0.01) to 0.34 (p = 0.11) [15]. The results of the second questionnaire were contrasted with medical and nursing notes documenting CAM use for inpatients [30], while findings from the third questionnaire were evaluated against CAM products found in patients’ homes [31]. The indeterminate rating was granted for each of these because correlation coefficients were not presented, not strong enough or not provided for all domains of the questionnaire.

All ratings for the four questionnaires [15]; [30]; [32]; [33] having assessed test-retest reliability were indeterminate because the majority did not present agreement coefficients such as Intraclass correlation coefficient or Kappa coefficients or confidence intervals. One of these questionnaires [15] presented variable scores of agreement depending on the item (Intraclass correlation coefficients between 0.52 to 0.78), which ranges from a negative to a positive rating, which amounted to an indeterminate rating. The same questionnaire also showed poor to questionable levels of internal consistency (α = 0.28 to 0.64). Inter-rater reliability was also indeterminate for the only questionnaire having assessed it (because the percentage of CAM use according to the different raters was reported but not the agreement between raters) [30].

According to the Cohen score, no questionnaire was considered to be a well-established one, two approached the level of a well-established assessment [33]; [34] and seven were promising assessments [18]; [19]; [21]; [22]; [24]; [28]; [35]. The remainder (n = 87) were either not described enough in publications (and the questionnaires not provided in the publication nor available upon request to the author) or their measurement properties were not reported at all. (See summary characteristics of the most promising questionnaires in Table S1).

Finally, among all included questionnaires, 19 were reported to have good readability/comprehension but only one of them using proper testing. This questionnaire was assessed using the Flesch-Kincaid grade reading level (score = 5.7), a test designed to indicate comprehension difficulty and based on two core measures (word and sentence length).

## Discussion

To the best of our knowledge, this systematic review is the first to formally aggregate the research evidence on the measurement properties of questionnaires assessing the use of CAM in pediatrics. This work will help to better understand the use of CAM in various pediatric clinical and research settings by informing future validation studies of CAM questionnaires. It will also help clinicians, researchers and the general public to better appraise the scientific literature on CAM in pediatrics.

The results of the current review suggest that many studies, mostly cross-sectional and conducted in the United States, have used CAM questionnaires to identify and understand the use of CAM among pediatric populations. However, CAM questionnaires varied greatly in terms of their characteristics. Some were disease-specific while others were generic, which may reflect that both types of instruments are seen as helpful in order to understand the use of CAM by researchers. Some succeed in targeting specific types of CAM used by patients with a certain condition while others have the advantage of assessing the use of CAM in many different conditions as well as the public at large, making it easier to compare use in different populations. Most of the questionnaires targeted parents and very few mentioned using a questionnaire specifically designed to gather children and adolescents self-report, which may be important to use in order to gather valid data about CAM use according to children. Such a self-report questionnaire may provide researchers with a valid tool to compare children’s and parents’ perceptions, which may lead to changes in the way health care providers discuss CAM with families. The high variability in the content of CAM questionnaires was also problematic and may explain great variability in use in the pediatric studies, such as in cancer care [2]. This may be explained by the fact that there is no consensus on a definition of CAM and the therapies that it encompasses as well as items that should be assessed in order to understand thoroughly the use of CAM [36]; [37]. Very few questionnaires were reported to have a completion time under 10 minutes, which might pose a problem if used in a research setting and routine clinical practice.

Studies included in our review were judged to be of poor methodological quality when evaluated by the COSMIN checklist since methods used to test measurement properties of CAM questionnaires were not described in enough detail to reach pre-defined criteria. Poor methodological quality of the studies may be explained by the fact that none of them were specifically designed to validate a CAM questionnaire, but rather sought to describe the use of CAM in specific populations. However, in order to do this, questionnaires still need to meet validity and reliability criteria and be described in a comprehensive manner.

None of the included CAM questionnaires met the Terwee criteria for measurement properties. The lack of thoroughly valid CAM questionnaires may be explained by the relative novelty of research in CAM compared to other fields and the small amount of funding provided to do such research, especially before the creation of various funding agencies in the early 1990’s such as the National Center for Complementary and Alternative Medicine (NCCAM) in the United States [38]. Moreover, funding agencies as well as the general public have been interested primarily in evaluating CAM use in specific populations possibly because it was most needed data to show the relevance of research in CAM. Furthermore, conducting validation studies takes time, money and energy, which may explain why researchers often engage in studies without using properly validated tools, even in fields other than CAM [39]. However, the fact that CAM questionnaires are not valid is a problem since results may not reflect the true use of CAM when such therapies are not clearly and comprehensively defined and presented to parents and children. An international CAM questionnaire was developed for adults and is currently being validated [40] but results may be difficult to extrapolate to children since perceptions concerning CAM may differ in these two populations [41]. Our results highlight the need for further research on defining and conceptualising CAM as well as developing valid questionnaires of its use, especially in pediatrics.

Using a research protocol similar to Terwee et al., helped to ensure credibility of our findings. However, the protocol as well as the COSMIN checklist and the Terwee score have been used for health status measures and not for CAM questionnaires. Because CAM questionnaires have been less extensively studied than health status measures, we encountered several difficulties in applying the same criteria. For example, the COSMIN checklist evaluates the methodological quality of validation studies, which were not conducted in CAM research and rendered parts of our assessment less pertinent. Furthermore, quality of reporting was very poor in CAM studies, which we tried to counteract by communicating with authors. Unfortunately, many did not reply and some replied without giving more details than their published work, which precludes us from knowing exactly how they proceeded to ensure their CAM questionnaires were adequate. Despite these limitations, we feel that currently available measures are inadequate and a new questionnaire is required.

In conclusion, our systematic review showed that many surveys have been conducted to document and understand the use of CAM in pediatrics without having properly validated their tools. Such tools, if valid, may prove to be useful in pediatric clinical settings to document the use of CAM as well as their perceived benefits and harms. This study highlights the need for research to define and conceptualise CAM as well as develop valid questionnaires of its use. Such tools may in turn lead to improved research and knowledge translation about CAM benefits and harms in clinical practice.

## Supporting Information

Figure S1 **PRISMA Flow Diagram of Included Articles.** Figure S1 presents a PRISMA flow diagram of articles included in the systematic review as well as the main reasons for rejection.
(TIFF)

Figure S2 **Domains Included in CAM Questionnaires.** Figure S2 presents the different domains that were included in the CAM questionnaires included in the systematic review.
(TIFF)

Table S1 **Characteristics of CAM Questionnaires** **Tested for Measurement Properties.**
(DOC)

Appendix S1 **Protocol.** Appendix S1 presents the protocol of the systematic review on measurement properties of CAM questionnaires.
(DOC)

Appendix S2 **Completed PRISMA checklist.** Appendix S2 presents the completed PRISMA checklist for the systematic review.
(PDF)

Appendix S3 **Search Strategy.** Appendix S3 presents the search strategy used to identify the CAM questionnaires.
(DOC)

Appendix S4 **COSMIN checklist and definitions of measurement properties.** Appendix S4 graphically presents the COSMIN checklist and defines the various measurement properties used to appraise the methodological quality of CAM studies.
(DOC)

Appendix S5 **Cohen criteria.** Appendix S5 presents the Cohen criteria used to evaluate the degree of testing of measurement properties of the included CAM questionnaires.
(DOC)

Appendix S6 **List of references.** Appendix S6 presents the list of references for included studies.
(DOC)
