# Supplementary figures and images for: Measurement Properties of Questionnaires Assessing Complementary and Alternative Medicine Use in Pediatrics: A Systematic Review
Source: PLoS One. 2012 Jun 29;7(6):e39611. doi: 10.1371/journal.pone.0039611 (PMC3387262; doi:10.1371/journal.pone.0039611)

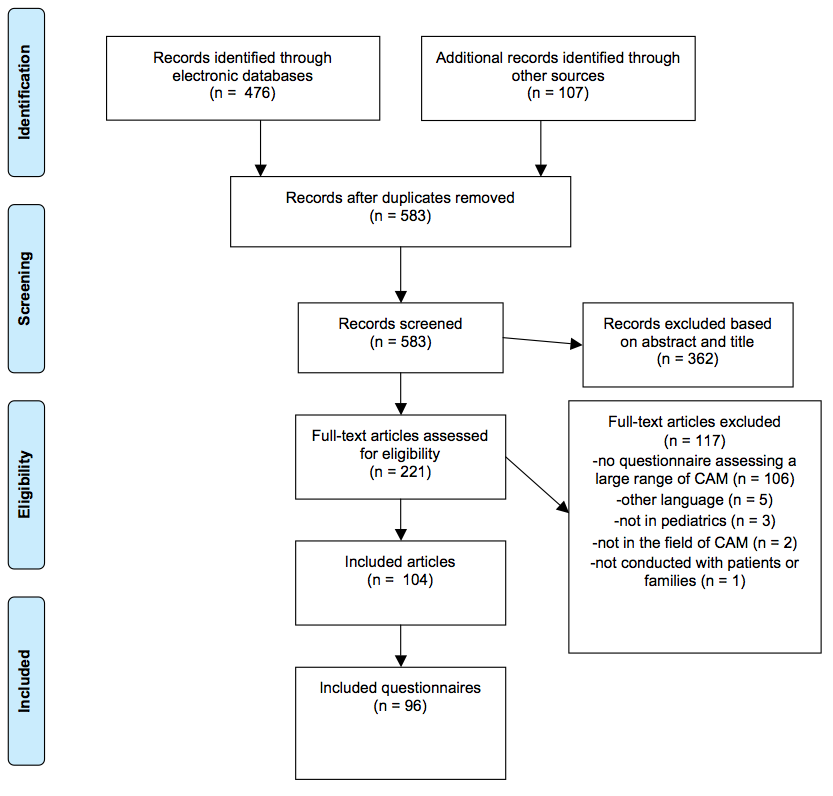

Supplement: Figure S1 — PRISMA Flow Diagram of Included Articles. Figure S1 presents a PRISMA flow diagram of articles included in the systematic review as well as the main reasons for rejection. (TIFF) [file pone.0039611.s001.tiff]

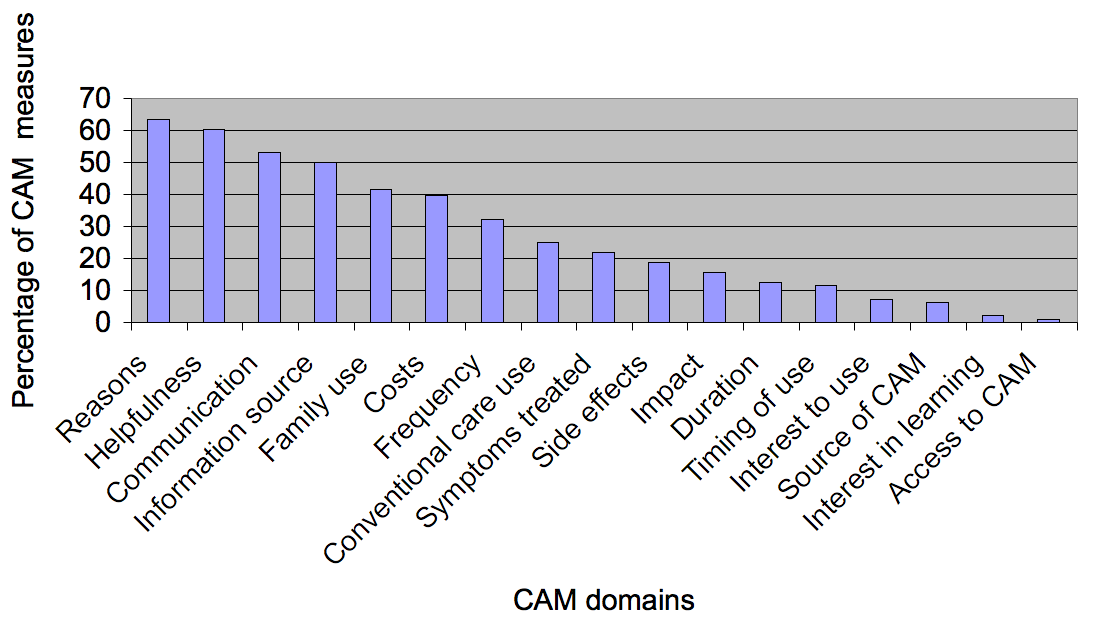

Supplement: Figure S2 — Domains Included in CAM Questionnaires. Figure S2 presents the different domains that were included in the CAM questionnaires included in the systematic review. (TIFF) [file pone.0039611.s002.tiff]
