# Supplementary material for: Measurement Properties of Questionnaires Assessing Complementary and Alternative Medicine Use in Pediatrics: A Systematic Review
Source: PLoS One. 2012 Jun 29;7(6):e39611. doi: 10.1371/journal.pone.0039611 (PMC3387262; doi:10.1371/journal.pone.0039611)
Supplement: Table S1 — Characteristics of CAM Questionnaires Tested for Measurement Properties. (DOC) [file pone.0039611.s003.doc]

## Table S1. **Characteristics of CAM Questionnaires Tested for Measurement Properties.**

| Author and year of study | Country | Health Condition targeted | Child Report | Time to administer* | Content/ criterion/ construct validity** | Test-retest/ inter-rater reliability/ internal consistency** | Cohen score‡ |
| --- | --- | --- | --- | --- | --- | --- | --- |
| Fernandez, 1998 | Canada | Cancer | No | ? | ? | ? | 1 |
| Simpson, 1998 and 2001 | UK | Pediatrics | No | ? | ? | ? | 0 |
| Bussing, 2002 | USA | ADHD | No | ? | ? | ? | 0 |
| Heuschkel, 2002 | USA/UK | IBD | No | - | ? | ? | 0 |
| Shenfield, 2002 | AUS | Asthma | Yes | ? | ? | ? | 0 |
| Hagen, 2003 | Canada | Rheumatology | Yes | ? | ? | ? | 1 |
| Loman, 2003 | USA | Pediatrics | No | ? | ? | ? | 0 |
| Braun, 2005 | USA | Pediatrics | Yes | - | ? | ? | 1 |
| Lim, 2005; Cincotta, 2006; Crawford, 2006; | UK/AUS | Pediatrics | Yes | - | ? | ? | 2 |
| Green, 2006 | International | Autism | No | ? | ? | ? | 0 |
| Smith, 2006 and MacLennan, 2006 | AUS | Pediatrics | Yes | ? | ? | ? | 2 |
| Dannemann, 2008 | Germany | Diabetes | ? | ? | ? | ? | 0 |
| Gerasimidis, 2008 | UK | IBD | No | - | ? | ? | 1 |
| Carlton, 2009 | UK | Pediatrics | No | ? | ? | ? | 1 |
| Hamidah, 2009 | Malaysia | Cancer | ? | ? | ? | ? | 0 |
| Post-White, 2009 | USA | Pediatrics | No | - | ? | ? | 1 |

*: + = less than 10 minutes, - = more than 10 minutes, ? = time to administer unknown

** : Rating according to the Terwee criteria: + = positive rating, ? = indeterminate rating, - = negative rating

### ‡ : Rating according to the Cohen criteria: 0 = not reaching criteria for promising instrument, 1= promising instrument, 2= approaching a well-established instrument, 3= well-established instrument

USA: United States of America, UK: United Kingdom, AUS: Australia, ADHD: Attention deficit-Hyperactivity disorder, IBD: Inflammatory bowel disease
