# Supplementary material for: Measurement Properties of Questionnaires Assessing Complementary and Alternative Medicine Use in Pediatrics: A Systematic Review
Source: PLoS One. 2012 Jun 29;7(6):e39611. doi: 10.1371/journal.pone.0039611 (PMC3387262; doi:10.1371/journal.pone.0039611)
Supplement: Appendix S1 — Protocol. Appendix S1 presents the protocol of the systematic review on measurement properties of CAM questionnaires. (DOC) [file pone.0039611.s004.doc]

University of Ottawa

**Appendix S1**

**Protocol**

Measurement Properties of Questionnaires Assessing Complementary and Alternative Medicine Use in Pediatrics

By

Karine Toupin April

Epidemiology and Community Medicine

Faculty of Medicine

April, 2011

Table of contents

Background [1](#__RefHeading___Toc318054651)

Review Questions [3](#__RefHeading___Toc318054652)

Criteria for considering studies for this review [4](#__RefHeading___Toc318054653)

Types of studies [4](#__RefHeading___Toc318054654)

Population targeted by the instruments [4](#__RefHeading___Toc318054655)

Instrument type [4](#__RefHeading___Toc318054656)

Comparison for outcomes of interest [4](#__RefHeading___Toc318054657)

Topic of interest [4](#__RefHeading___Toc318054658)

Review methods [5](#__RefHeading___Toc318054659)

Team members [5](#__RefHeading___Toc318054660)

Search strategy [5](#__RefHeading___Toc318054661)

Identification of studies [6](#__RefHeading___Toc318054662)

Data extraction [7](#__RefHeading___Toc318054663)

Quality assessment [8](#__RefHeading___Toc318054664)

Data synthesis [9](#__RefHeading___Toc318054665)

Data analysis [9](#__RefHeading___Toc318054666)

Potential conflict of interest [10](#__RefHeading___Toc318054667)

Sources of support [10](#__RefHeading___Toc318054668)

# Background

Scientific literature and the media suggest an increased interest in complementary and alternative medicine (CAM) among the general public and a tendency towards an increased use (1-3), particularly in children with chronic diseases (4).

CAM may include many types of therapies or products that are currently not considered to be part of conventional medicine and are classified in four domains by the National Center for Complementary and Alternative Medicine (NCCAM) (5). These domains of CAM are: 1) natural products (e.g. vitamins, minerals, dietary supplements, probiotics); 2) mind-body medicine (e.g. meditation, yoga, acupuncture, deep-breathing exercises, guided imagery, tai chi); 3) manipulative and body-based practices (e.g. spinal manipulation, massage therapy); and 4) other CAM practices (e.g. movement therapies, traditional healers, energy therapies). Even though the NCCAM definition is widely used, there is no consensus on the definition of CAM (6) or the types of therapies or products that should be considered as such (7), which makes it difficult to collect data in a standardized manner in both research and clinical settings.

The increasing interest in CAM stresses the importance for health care providers to initiate a dialogue about CAM use in order to assess its impact on their lives and to provide the most up-to-date evidence-based advice on risks and benefits of these therapies. However, there seems to be a lack of standardized methods and validated questionnaires to assess patients’ use and perceptions of CAM in this population (6). To date, no systematic review has assessed the extent of measurement properties of CAM questionnaires in pediatrics.

# The objective of this systematic review was to critically appraise and summarize the research evidence on the measurement properties of questionnaires assessing the use of CAM in pediatrics.

# Review Questions

Have questionnaires assessing the use of complementary and alternative medicine in pediatrics been thoroughly validated?

# Criteria for considering studies for this review

## Types of questionnaires and studies

We will include questionnaires that seek to assess the prevalence of a wide range of CAM use (more than one type or one sub-category of CAM), such as products and services provided by CAM practitioners. All types of studies (e.g. psychometric studies, prevalence studies and clinical trials) will be included if they report using such a questionnaire and are reported in English or French language articles. Grey literature, such as abstracts published in scientific journals or proceedings of conferences will be excluded due to lack of information concerning questionnaires used.

## Population targeted by the instruments

Questionnaires targeting children aged 0-18 years old. Questionnaires could be used with parents or children themselves but need to assess children’s CAM use.

## Instrument type

Questionnaires

## Types of outcomes of interest

Primary

Determine the measurement properties of CAM questionnaires and the methodological quality of included studies

Secondary

Determine the content of CAM questionnaires: CAM use and possibly other related constructs (e.g. perceived effectiveness of CAM, reasons for CAM use, cost of CAM, communication with health practitioners about CAM)

# Review methods

## Team members

The team will be comprised of methodologists with a knowledge of CAM (Dr. Heather Boon, Dr. David Moher, Dr. Sunita Vohra and Dr. Karine Toupin April), clinicians with research methodology knowledge (Dr. Peter Tugwell, Dr. Ciaran Duffy, Dr. Jennifer Stinson), a graduate student (Ani Byrne), a research assistant (Meghan White) and a medical student (Bharbhoor Dhaliwal). Several members of this team are experts in the conduct of systematic reviews. We are also collaborating with three librarians (Tamara Rader, Jessie McGowan and Soleil Surette).

## Search strategy

An electronic search strategy (See appendix) will be developed by a librarian (TR) and refined using the peer review of electronic search strategies (PRESS) checklist (8). The search strategy will be implemented in the following electronic databases: MEDLINE (1950 to week 12 2011), Healthstar, Cochrane CENTRAL, AMED (1985 to 2011), PsycINFO, The Cochrane Collaboration’s Methodology Register, EMBASE (1980 to 2011) and the Health and Psychosocial Instruments (HaPI) database (1985 to the last week of March 2011). Conference websites, scientific journals and experts in the field of CAM will also be consulted in order to identify relevant publications of CAM questionnaires.

The following search strategy has been formulated with the help of two librarians. It will be used for OVID MEDLINE but will be modified for each database:

Database: Ovid MEDLINE(R) In-Process & Other Non-Indexed Citations and Ovid MEDLINE(R) <1950 to Present>

Search Strategy:

--------------------------------------------------------------------------------

1 exp "Outcome Assessment (Health Care)"/ (417450)

2 exp Clinical Trials as Topic/mt (10127)

3 Psychometrics/mt (2407)

4 exp "Reproducibility of Results"/ (177354)

5 (Sensitivity and Specificity).mp. (262151)

6 correlation.mp. (423866)

7 Validation Studies/ (39995)

8 Validation.tw. (56130)

9 Validation Studies as Topic/ (215)

10 Face validity.tw. (938)

11 Content validity.tw. (1893)

12 Construct validity.tw. (6437)

13 concurrent validity.tw. (2443)

14 Convergent validity.tw. (1809)

15 Discriminant validity.tw. (2026)

16 or/1-15 (1221646)

17 *Questionnaires/ (17798)

18 tool.mp. or toolkit.tw. (165171)

19 Checklist.tw. (10942)

20 instrument.tw. (53683)

21 survey.tw. (220546)

22 Evaluation.tw. (585477)

23 *Evaluation Studies as Topic/ (5615)

24 Performance measures.mp. (2570)

25 or/17-24 (999017)

26 exp Complementary Therapies/ (131463)

27 Complementary medicine.mp. (1246)

28 Complementary health$.tw. (108)

29 integrated health.mp. (1161)

30 Medicine, Chinese Traditional/ (7170)

31 integrated medicine.mp. (88)

32 integrated therap$.tw. (181)

33 Alternative medicine.mp. (3979)

34 Alternative health$.tw. (457)

35 Alternative Therap$.mp. (5500)

36 or/26-35 (138321)

37 *Child/ (51857)

38 children.mp. (566702)

39 exp Pediatrics/ (34455)

40 paediatric.tw. (23132)

41 exp Parents/ (51374)

42 Legal Guardians/ or guardian.mp. (2781)

43 or/37-42 (670965)

44 25 and 36 and 43 and 16 (146)

45 from 44 keep 1-146 (146)

We will search websites such as the National Center for Complementary and Alternative Medicine (www.nccam.nih.gov/clinicaltrials/), the Chair of Complementary Medicine at the Peninsula Medical School, Universities of Exeter and Plymouth (www.pms.ac.uk/compmed/), and the National Institutes of Health’s Clinical Trials Database ([www.clinicaltrials.gov](http://www.clinicaltrials.gov/)). We will also do a manual search of major journals in CAM and will correspond with experts in this field in order to retrieve studies that are not yet indexed in databases.

Results from our search will be exported into Reference Manager.

##

## Identification of studies

Two members of the team (KTA and AB or BD) will independently screen the titles, abstracts and key words of the studies identified from the search and apply the eligibility criteria. Full text articles will be obtained for relevant studies. Each reviewer will then decide independently which articles should be included in the systematic review. Should there be any disagreements, another member of the team will resolve the issue (JS or DM).

A flow PRISMA diagram will illustrate which studies will be included in the review or rejected.

## Data extraction

Data from included studies will be independently extracted by two members of the team (KTA and MW or AB) using the data abstraction forms and then entered into an excel spread sheet. The data extraction forms will be pilot-tested before being used. If some data from the studies is insufficient or missing, we will attempt to obtain this information from the authors by personal communication (up to three e-mails).

The following data will be extracted:

- Characteristics of the studies (study design, objectives, geographical location, target population) and the questionnaires (purpose, child and/or proxy report, content, time to complete).
- Information pertaining to the methodological quality of studies and measurement properties of questionnaires
  - Methodological quality of studies reporting each CAM questionnaire using the COSMIN checklist
  - Measurement properties of the CAM questionnaires themselves were assessed using the Terwee and Cohen criteria.

In the event of disagreement between reviewers, a third reviewer will resolve the issue.

## Quality assessment

The methodological quality of studies reporting each CAM questionnaire was assessed using the COSMIN checklist (9-12). The measurement properties of the CAM questionnaires themselves were assessed using the Terwee and Cohen criteria (13;14). Assessments were independently performed by two members of the team (KTA and MW or AB). When data were missing from a study, corresponding authors were e-mailed in an effort to obtain questionnaires and additional information. Authors were also e-mailed at the end of our systematic review in order to confirm results.

## Data synthesis

We will describe the characteristics of the included studies, the questionnaires and the patients in several tables. By assessing the quality of included studies, it will be possible to synthesize the data extracted into a coherent summary.

## Data analysis

We will do a narrative summary of the results. The data will be analyzed using content analysis. Descriptive statistics were used to describe the characteristics of the studies and questionnaires.

# Potential conflict of interest

There is a potential conflict of interest since the main author of this systematic review (KTA) has published articles that mention a CAM questionnaire. However, to minimize this potential bias, another member of the research team will be involved in the identification of studies, the extraction of data and the quality assessment.

# Sources of support

External sources of support:

This project was funded by the Canadian Arthritis Network.

Internal sources of support:

Dr. Karine Toupin April has been supported by a postdoctoral fellowship from the Canadian Arthritis Network, The Arthritis Society and the Canadian Institutes of Health Research. Dr. Moher holds a University Research Chair. Dr. Stinson’s research is supported by a Ministry of Health and Long-term Care Career Scientist Award. Dr. Ciarán M Duffy holds an Endowed Chair, Department of Paediatrics, University of Ottawa. Dr. Sunita Vohra receives salary support as an Alberta Innovates-Health Solutions Health Scholar. Dr. Peter Tugwell holds a Canada Research Chair in Health Equity.
