# Supplementary material for: Measurement Properties of Questionnaires Assessing Complementary and Alternative Medicine Use in Pediatrics: A Systematic Review
Source: PLoS One. 2012 Jun 29;7(6):e39611. doi: 10.1371/journal.pone.0039611 (PMC3387262; doi:10.1371/journal.pone.0039611)
Supplement: Appendix S3 — Search Strategy. Appendix S3 presents the search strategy used to identify the CAM questionnaires. (DOC) [file pone.0039611.s006.doc]

**Appendix S3**

**Search Strategy**

Database: Ovid MEDLINE(R) In-Process & Other Non-Indexed Citations and Ovid MEDLINE(R) <1950 to Present>

Search Strategy:

--------------------------------------------------------------------------------

1 exp "Outcome Assessment (Health Care)"/ (417450)

2 exp Clinical Trials as Topic/mt (10127)

3 Psychometrics/mt (2407)

4 exp "Reproducibility of Results"/ (177354)

5 (Sensitivity and Specificity).mp. (262151)

6 correlation.mp. (423866)

7 Validation Studies/ (39995)

8 Validation.tw. (56130)

9 Validation Studies as Topic/ (215)

10 Face validity.tw. (938)

11 Content validity.tw. (1893)

12 Construct validity.tw. (6437)

13 concurrent validity.tw. (2443)

14 Convergent validity.tw. (1809)

15 Discriminant validity.tw. (2026)

16 or/1-15 (1221646)

17 *Questionnaires/ (17798)

18 tool.mp. or toolkit.tw. (165171)

19 Checklist.tw. (10942)

20 instrument.tw. (53683)

21 survey.tw. (220546)

22 Evaluation.tw. (585477)

23 *Evaluation Studies as Topic/ (5615)

24 Performance measures.mp. (2570)

25 or/17-24 (999017)

26 exp Complementary Therapies/ (131463)

27 Complementary medicine.mp. (1246)

28 Complementary health$.tw. (108)

29 integrated health.mp. (1161)

30 Medicine, Chinese Traditional/ (7170)

31 integrated medicine.mp. (88)

32 integrated therap$.tw. (181)

33 Alternative medicine.mp. (3979)

34 Alternative health$.tw. (457)

35 Alternative Therap$.mp. (5500)

36 or/26-35 (138321)

37 *Child/ (51857)

38 children.mp. (566702)

39 exp Pediatrics/ (34455)

40 paediatric.tw. (23132)

41 exp Parents/ (51374)

42 Legal Guardians/ or guardian.mp. (2781)

43 or/37-42 (670965)

44 25 and 36 and 43 and 16 (146)

45 from 44 keep 1-146 (146)
