# Supplementary material for: Measurement Properties of Questionnaires Assessing Complementary and Alternative Medicine Use in Pediatrics: A Systematic Review
Source: PLoS One. 2012 Jun 29;7(6):e39611. doi: 10.1371/journal.pone.0039611 (PMC3387262; doi:10.1371/journal.pone.0039611)
Supplement: Appendix S4 — COSMIN checklist and definitions of measurement properties. Appendix S4 graphically presents the COSMIN checklist and defines the various measurement properties used to appraise the methodological quality of CAM studies. (DOC) [file pone.0039611.s007.doc]

**Appendix S4**

**COSMIN checklist and definitions of measurement properties**

**
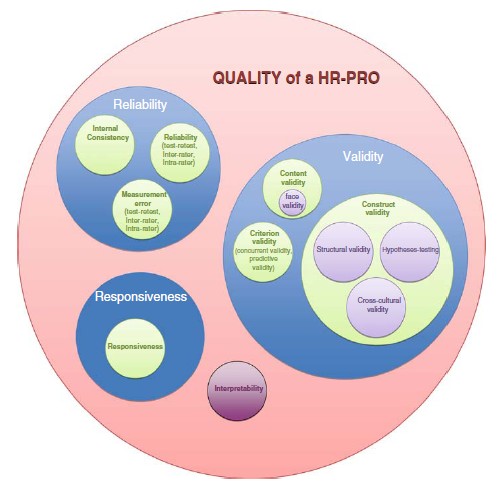
**

Figure originally published in: Mokkink LB, Terwee CB, Patrick DL et al. The COSMIN checklist for assessing the methodological quality of studies on measurement properties of health status measurement instruments: an international Delphi study. Qual Life Res 2010; 19(4):539-549. Reproduced with permission from Springer Publishing©.

Definitions:

Internal consistency: The degree of the interrelatedness among the items.

Reliability The proportion of the total variance in the measurements which is because of “true” differences among patients.

Measurement error: The systematic and random error of a patient’s score that is not attributed to true changes in the construct to be measured.

Validity: The degree to which an health-related patient-reported outcomes (HR-PRO) instrument measures the construct(s) it purports to measure.

Content validity: The degree to which the content of an HR-PRO instrument is an adequate reflection of the construct to be measured.

Face validity: The degree to which (the items of) an HR-PRO instrument indeed looks as though they are an adequate reflection of the construct to be measured.

Construct validity: The degree to which the scores of an HR-PRO instrument are consistent with hypotheses (for instance with regard to internal relationships, relationships to scores of other instruments, or differences between relevant groups) based on the assumption that the HR-PRO instrument validly measures the construct to be measured.

Structural validity: The degree to which the scores of an HR-PRO instrument are an adequate reflection of the dimensionality of the construct to be measured.

Cross-cultural validity: The degree to which the performance of the items on a translated or culturally adapted HR-PRO instrument are an adequate reflection of the performance of the items of the original version of the HR-PRO Instrument.

Criterion validity: The degree to which the scores of an HR-PRO instrument are an adequate reflection of a “gold standard”.

Responsiveness: The ability of an HR-PRO instrument to detect change over time in the construct to be measured.

Interpretability (characteristic but not a measurement property): The degree to which one can assign qualitative meaning - that is, clinical or commonly understood connotations - to an instrument’s quantitative scores or change in scores.

Definitions originally published in: Mokkink LB, Terwee CB, Patrick DL et al. The COSMIN study reached international consensus on taxonomy, terminology, and definitions of measurement properties for health-related patient-reported outcomes. J Clin Epidemiol 2010; 63(7):737-745. Reproduced with permission from Elsevier Limited ©.
