# Supplementary material for: Measurement Properties of Questionnaires Assessing Complementary and Alternative Medicine Use in Pediatrics: A Systematic Review
Source: PLoS One. 2012 Jun 29;7(6):e39611. doi: 10.1371/journal.pone.0039611 (PMC3387262; doi:10.1371/journal.pone.0039611)
Supplement: Appendix S5 — Cohen criteria. Appendix S5 presents the Cohen criteria used to evaluate the degree of testing of measurement properties of the included CAM questionnaires. (DOC) [file pone.0039611.s008.doc]

**Appendix S5**

**Cohen criteria**

**
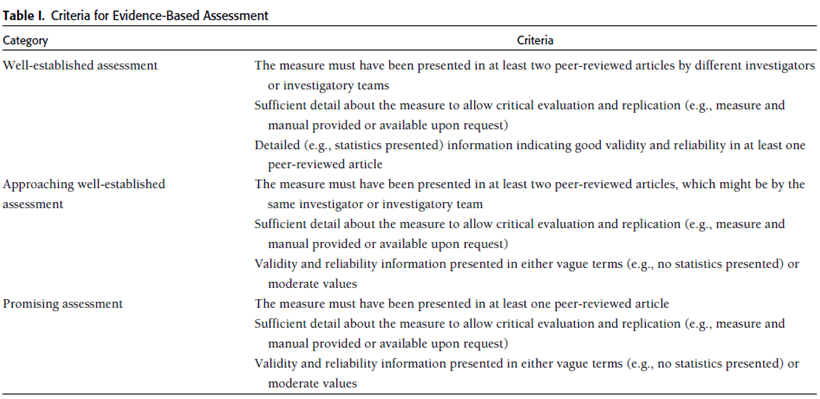
**

Originally published in: Cohen LL, La Greca AM, Blount RL, Kazak AE, Holmbeck GN, Lemanek KL. Introduction to special issue: Evidence-based assessment in pediatric psychology. J Pediatr Psychol 2008; 33(9):911-915. Reproduced with permission from Oxford University Press ©.
