# Supplementary material for: Measurement Properties of Questionnaires Assessing Complementary and Alternative Medicine Use in Pediatrics: A Systematic Review
Source: PLoS One. 2012 Jun 29;7(6):e39611. doi: 10.1371/journal.pone.0039611 (PMC3387262; doi:10.1371/journal.pone.0039611)
Supplement: Appendix S6 — List of references. Appendix S6 presents the list of references for included studies. (DOC) [file pone.0039611.s009.doc]

**Appendix S6**

**List of references**

[1] Ang JY, Ray-Mazumder S, Nachman SA, Rongkavilit C, Asmar BI et al. Use of complementary and alternative medicine by parents of children with HIV infection and asthma and well children. South Med J 2005; 98(9):869-875.

[2] Armishaw J, Grant CC. Use of complementary treatment by those hospitalised with acute illness. Arch Dis Child 1999; 81(2):133-137.

[3] Babayigit A, Olmez D, Karaman O, Uzuner N. Complementary and alternative medicine use in Turkish children with bronchial asthma. J Altern Complement Med 2008; 14(7):797-799.

[4] Braganza S, Ozuah PO, Sharif I. The use of complementary therapies in inner-city asthmatic children. J Asthma 2003; 40(7):823-827.

[5] Braun CA, Bearinger LH, Halcon LL, Pettingell SL. Adolescent use of complementary therapies. J Adolesc Health 2005; 37(1):76.

[6] Carlton P, Johnson I, Cunliffe C. Factors influencing parents' decisions to choose chiropractic care for their children in the UK. Clinical Chiropractic 2009; 12:11-22.

[7] Chan E, Rappaport LA, Kemper KJ. Complementary and alternative therapies in childhood attention and hyperactivity problems. J Dev Behav Pediatr 2003; 24(1):4-8.

[8] Cheshire A, Powell L, Barlow J. Use of complementary and alternative medicine for children with brain injury in the United Kingdom. J Altern Complement Med 2007; 13(7):703-704.

[9] Dannemann K, Hecker W, Haberland H, Herbst A, Galler A et al. Use of complementary and alternative medicine in children with type 1 diabetes mellitus - prevalence, patterns of use, and costs. Pediatr Diabetes 2008; 9(3 Pt 1):228-235.

[10] Davis MP, Darden PM. Use of complementary and alternative medicine by children in the United States. Arch Pediatr Adolesc Med 2003; 157(4):393-396.

[11] Day AS. Use of complementary and alternative therapies and probiotic agents by children attending gastroenterology outpatient clinics. J Paediatr Child Health 2002; 38(4):343-346.

[12] Day AS, Whitten KE, Bohane TD. Use of complementary and alternative medicines by children and adolescents with inflammatory bowel disease. J Paediatr Child Health 2004; 40(12):681-684.

[13] Feldman DE, Duffy C, De Civita M, Malleson P, Philibert L et al. Factors associated with the use of complementary and alternative medicine in juvenile idiopathic arthritis. Arthritis Rheum 2004; 51(4):527-532.

[14] Friedman T, Slayton WB, Allen LS, Pollock BH, Dumont-Driscoll M et al. Use of alternative therapies for children with cancer. Pediatrics 1997; 100(6):E1.

[15] Genc RE, Senol S, Turgay AS, Kantar M. Complementary and alternative medicine used by pediatric patients with cancer in western Turkey. Oncol Nurs Forum 2009; 36(3):E159-E164.

[16] Gerasimidis K, McGrogan P, Hassan K, Edwards CA. Dietary modifications, nutritional supplements and alternative medicine in paediatric patients with inflammatory bowel disease. Aliment Pharmacol Ther 2008; 27(2):155-165.

[17] Goin-Kochel RP, Mackintosh VH, Myers BJ. How many doctors does it take to make an autism spectrum diagnosis? Autism 2006; 10(5):439-451.

[18] Golomb MR, Hune S, MacGregor DL, deVeber GA. Alternative therapy use by Chinese-Canadian children with stroke and cerebrovascular disease. J Child Neurol 2003; 18(10):714-717.

[19] Gomez-Martinez R, Tlacuilo-Parra A, Garibaldi-Covarrubias R. Use of complementary and alternative medicine in children with cancer in Occidental, Mexico. Pediatr Blood Cancer 2007; 49(6):820-823.

[20] Green VA, Pituch KA, Itchon J, Choi A, O'Reilly M et al. Internet survey of treatments used by parents of children with autism. Res Dev Disabil 2006; 27(1):70-84.

[21] Gross-Tsur V, Lahad A, Shalev RS. Use of complementary medicine in children with attention deficit hyperactivity disorder and epilepsy. Pediatr Neurol 2003; 29(1):53-55.

[22] Hamidah A, Rustam ZA, Tamil AM, Zarina LA, Zulkifli ZS et al. Prevalence and parental perceptions of complementary and alternative medicine use by children with cancer in a multi-ethnic Southeast Asian population. Pediatr Blood Cancer 2009; 52(1):70-74.

[23] Harrington JW, Rosen L, Garnecho A, Patrick PA. Parental perceptions and use of complementary and alternative medicine practices for children with autistic spectrum disorders in private practice. J Dev Behav Pediatr 2006; 27(2 Suppl):S156-S161.

[24] Hughes R, Ward D, Tobin AM, Keegan K, Kirby B. The use of alternative medicine in pediatric patients with atopic dermatitis. Pediatr Dermatol 2007; 24(2):118-120.

[25] Jean D, Cyr C. Use of complementary and alternative medicine in a general pediatric clinic. Pediatrics 2007; 120(1):e138-e141.

[26] Johnston GA, Bilbao RM, Graham-Brown RA. The use of complementary medicine in children with atopic dermatitis in secondary care in Leicester. Br J Dermatol 2003; 149(3):566-571.

[27] Karadeniz C, Pinarli FG, Oguz A, Gursel T, Canter B. Complementary/alternative medicine use in a pediatric oncology unit in Turkey. Pediatr Blood Cancer 2007; 48(5):540-543.

[28] Kelly KM, Jacobson JS, Kennedy DD, Braudt SM, Mallick M et al. Use of unconventional therapies by children with cancer at an urban medical center. J Pediatr Hematol Oncol 2000; 22(5):412-416.

[29] Laengler A, Spix C, Seifert G, Gottschling S, Graf N et al. Complementary and alternative treatment methods in children with cancer: A population-based retrospective survey on the prevalence of use in Germany. Eur J Cancer 2008; 44(15):2233-2240.

[30] Lim A, Cranswick N, Skull S, South M. Survey of complementary and alternative medicine use at a tertiary children's hospital. J Paediatr Child Health 2005; 41(8):424-427.

[31] Lim J, Wong M, Chan MY, Tan AM, Rajalingam V et al. Use of complementary and alternative medicine in paediatric oncology patients in Singapore. Ann Acad Med Singapore 2006; 35(11):753-758.

[32] Lin YC, Bioteau AB, Ferrari LR, Berde CB. The use of herbs and complementary and alternative medicine in pediatric preoperative patients. J Clin Anesth 2004; 16(1):4-6.

[33] Loman DG. The use of complementary and alternative health care practices among children. J Pediatr Health Care 2003; 17(2):58-63.

[34] Low E, Murray DM, O'Mahony O, O'B HJ. Complementary and alternative medicine use in Irish paediatric patients. Ir J Med Sci 2008; 177(2):147-150.

[35] Madsen H, Andersen S, Nielsen RG, Dolmer BS, Host A et al. Use of alternative therapies among pediatric patients. Ugeskr Laeger 2003; 165(43):4108-4112.

[36] Markowitz JE, Mamula P, delRosario JF, Baldassana RN, Lewis JD et al. Patterns of complementary and alternative medicine use in a population of pediatric patients with inflammatory bowel disease. Inflamm Bowel Dis 2004; 10(5):599-605.

[37] McCarty RL, Weber WJ, Loots B, Breuner CC, Vander Stoep A et al. Complementary and alternative medicine use and quality of life in pediatric diabetes. J Altern Complement Med 2010; 16(2):165-173.

[38] McCurdy EA, Spangler JG, Wofford MM, Chauvenet AR, McLean TW. Religiosity is associated with the use of complementary medical therapies by pediatric oncology patients. J Pediatr Hematol Oncol 2003; 25(2):125-129.

[39] Menniti-Ippolito F, Gargiulo L, Bologna E, Forcella E, Raschetti R. Use of unconventional medicine in Italy: a nation-wide survey. Eur J Clin Pharmacol 2002; 58(1):61-64.

[40] Molassiotis A, Cubbin D. 'Thinking outside the box': complementary and alternative therapies use in paediatric oncology patients. Eur J Oncol Nurs 2004; 8(1):50-60.

[41] Mottonen M, Uhari M. Use of micronutrients and alternative drugs by children with acute lymphoblastic leukemia. Med Pediatr Oncol 1997; 28(3):205-208.

[42] Nathanson I, Sandler E, Ramirez-Garnica G, Wiltrout SA. Factors influencing complementary and alternative medicine use in a multisite pediatric oncology practice. J Pediatr Hematol Oncol 2007; 29(10):705-708.

[43] Neuhouser ML, Patterson RE, Schwartz SM, Hedderson MM, Bowen DJ et al. Use of alternative medicine by children with cancer in Washington state. Prev Med 2001; 33(5):347-354.

[44] Ozturk C, Karayagiz G. Exploration of the use of complementary and alternative medicine among Turkish children. J Clin Nurs 2008; 17(19):2558-2564.

[45] Pitetti R, Singh S, Hornyak D, Garcia SE, Herr S. Complementary and alternative medicine use in children. Pediatr Emerg Care 2001; 17(3):165-169.

[46] Reznik M, Ozuah PO, Franco K, Cohen R, Motlow F. Use of complementary therapy by adolescents with asthma. Arch Pediatr Adolesc Med 2002; 156(10):1042-1044.

[47] Rouster-Stevens K, Nageswaran S, Arcury TA, Kemper KJ. How do parents of children with juvenile idiopathic arthritis (JIA) perceive their therapies? BMC Complement Altern Med 2008; 8:25.

[48] Samdup DZ, Smith RG, Song SI. The use of complementary and alternative medicine in children with chronic medical conditions. Am J Phys Med Rehabil 2006; 85(10):842-846.

[49] Sawni-Sikand A, Schubiner H, Thomas RL. Use of complementary/alternative therapies among children in primary care pediatrics. Ambul Pediatr 2002; 2(2):99-103.

[50] Shakeel M, Little SA, Bruce J, Ah-See KW. Use of complementary and alternative medicine in pediatric otolaryngology patients attending a tertiary hospital in the UK. Int J Pediatr Otorhinolaryngol 2007; 71(11):1725-1730.

[51] Sidora-Arcoleo K, Yoos HL, McMullen A, Kitzman H. Complementary and alternative medicine use in children with asthma: prevalence and sociodemographic profile of users. J Asthma 2007; 44(3):169-175.

[52] Singer L, Karakis I, Ivri L, Gross M, Bolotin A et al. The characteristics of complementary and alternative medicine use by parents of asthmatic children in Southern Israel. Acta Paediatr 2007; 96(11):1693-1697.

[53] Sinha D, Efron D. Complementary and alternative medicine use in children with attention deficit hyperactivity disorder. J Paediatr Child Health 2005; 41(1-2):23-26.

[54] Smith C, Eckert K. Prevalence of complementary and alternative medicine and use among children in South Australia. J Paediatr Child Health 2006; 42(9):538-543.

[55] Soo I, Mah JK, Barlow K, Hamiwka L, Wirrell E. Use of complementary and alternative medical therapies in a pediatric neurology clinic. Can J Neurol Sci 2005; 32(4):524-528.

[56] Toupin-April K, Feldman DE, Zunzunegui MV, Descarreaux M, Malleson P et al. Is complementary and alternative healthcare use associated with better outcomes in children with juvenile idiopathic arthritis? J Rheumatol 2009; 36(10):2302-2307.

[57] Weyl Ben Arush M, Geva H, Ofir R, Mashiach T, Uziel R et al. Prevalence and characteristics of complementary medicine used by pediatric cancer patients in a mixed western and middle-eastern population. J Pediatr Hematol Oncol 2006; 28(3):141-146.

[58] Wilson K, Dowson C, Mangin D. Prevalence of complementary and alternative medicine use in Christchurch, New Zealand: children attending general practice versus paediatric outpatients. N Z Med J 2007; 120(1251):U2464.

[59] Wong HH, Smith RG. Patterns of complementary and alternative medical therapy use in children diagnosed with autism spectrum disorders. J Autism Dev Disord 2006; 36(7):901-909.

[60] Wong VC. Use of complementary and alternative medicine (CAM) in autism spectrum disorder (ASD): comparison of Chinese and western culture (Part A). J Autism Dev Disord 2009; 39(3):454-463.

[61] Zebracki K, Holzman K, Bitter KJ, Feehan K, Miller ML. Brief report: use of complementary and alternative medicine and psychological functioning in Latino children with juvenile idiopathic arthritis or arthralgia. J Pediatr Psychol 2007; 32(8):1006-1010.

[62] Zuzak TJ, Zuzak-Siegrist I, Rist L, Staubli G, Simoes-Wust AP. Medicinal systems of complementary and alternative medicine: a cross-sectional survey at a pediatric emergency department. J Altern Complement Med 2010; 16(4):473-479.

[63] Martel D, Bussières J-F, Théorêt Y, Lebel D, Kish S et al. Use of Alternative and Complementary Therapies in Children with Cancer. Pediatrc Blood Cancer 2005; 44:660-668.

[64] Breuner CC, Barry PJ, Kemper KJ. Alternative Medicine Use by Homeless Youth. Arch Pediatr Adolesc Med 1998; 152:1071-1075.

[65] Pachter LM, Sumner T, Fontan A, Sneed M, Bernstein BA. Home-Based Therapies for the Common Cold among European American and Ethnic Minority Families. Archives of Pediatric and adolescent medicine 1998; 152:1083-1088.

[66] Moenkhoff M, Baenziger O, Fischer J, Fanconi S. Parental attitude towards alternative medicine in the paediatric intensive care unit. Eur J Pediatr 1998; 158:12-17.

[67] Fernandez C, Stutzer C, MacWilliam L, Fryer C. Alternative and complementary therapy use in pediatric oncology patients in British Columbia: Prevalence and reasons for use and nonuse. Journal of clinical oncology 1998; 16(4):1279-1286.

[68] Simpson N, Pearce A, Finlay F, Lenton S. The use of complementary medicine in paediatric outpatient clinics. Ambulatory Child Health 1998; 3:351-356.

[69] Andrews L, Lokuge S, Sawyer M, Lillywhite L, Kennedy D et al. The use of alternative therapies by children with asthma: A brief report. J Paediatr Child Health 1998; 34:131-134.

[70] Stubberfield TG, Wray JA, Parry TS. Utilization of Alternative Therapies in Attention-Deficit Hyperactivity Disorder. J Paediatr Child Health 1999; 35:450-453.

[71] Simpson N, Roman K. Complementary medicine use in children: extent and reasons. A population-based study. British Journal of General Practice 2001; 51(472):914-916.

[72] Bold J, Leis A. Unconventional therapy use among children with cancer in Saskatchewan. Journal of Pediatric Oncology Nursing 2001 Jan-Feb;18(1):16-25 2001.

[73] Matthees B, Anantachoti P, Kreitzer MJ, Savik K, Hertz MI et al. Use of complementary therapies, adherence, and quality of life in lung transplant recipients. Heart Lung 2001; 30:258-268.

[74] Ottolini MC, Hamburger EK, Loprieato JO, Coleman RH, Sachs HC et al. Complementary and alternative medicine use among children in the Washington, DC area. Ambulatory Pediatrics 2001; 1(2):122-125.

[75] Bussing R, Zima BT, Gary FA, Garvan CW. Use of complementary and alternative medicine for symptoms of attention-deficit hyperactivity disorder. Psychiatric Services 2002; 53(9):1096-1102.

[76] Shenfield G, Lim E, Allen H. Survey of the use of complementary medicines and therapies in children with asthma. Journal of Paediatrics & Child Health 2002; 38(3):252-257.

[77] Fong DPS, Fong LKS. Usage of complementary medicine among children. Australian Family Physician 2002; 31(4):388-391.

[78] Heuschkel R, Afzal N, Wuerth A, Zurakowski D, Leichtner A, et al. Complementary medicine use in children and young adults with inflammatory bowel disease. American Journal of Gastroenterology 2002; 97(2):382-388.

[79] Sanders H, Davis MF, Duncan B, Meaney FJ, Haynes J et al. Use of complementary and alternative medical therapies among children with special health care needs in southern Arizona. Pediatrics 2003; 111:584-587.

[80] Orhan F, Sekerel BE, Kocabas CN, Sackesen C, Adalioglu G et al. Complementary and alternative medicine in children with asthma.[see comment]. Annals of Allergy, Asthma, & Immunology 2003; 90(6):611-615.

[81] Gagnon EM, Recklitis CJ. Parents' decision-making preferences in pediatric oncology: the relationship to health care involvement and complementary therapy use. Psycho-Oncology 2003; 12(5):442-452.

[82] Yussman S, Ryan S, Auinger P, Weitzman M. Visits to Complementary and Alternative Medicine Providers by Children and Adolescents in the United States. Ambulatory Pediatrics 2004; 4:429-435.

[83] Losier A, Taylor B, Fernandez CV. Use of alternative therapies by patients presenting to a pediatric emergency department. Journal of Emergency Medicine 2005; 28(3):267-271.

[84] Baron SE, Goodwin RG, Nicolau N, Blackford S, Goulden V. Use of complementary medicine among outpatients with dermatologic conditions within Yorkshire and South Wales, United Kingdom. J Am Acad Dematol 2005; 52:589-594.

[85] Crawford NW, Cincotta DR, Lim A, Powell CVE. A cross-sectional survey of complementary and alternative medicine use by children and adolescents attending the University Hospital of Wales. BMC Complementary and Alternative Medicine 2006; 6(16).

[86] Wilson KM, Klein JD, Sesselberg TS, Yussman SM, Markow DB et al. Use of complementary medicine and dietary supplements among U.S. adolescents. Journal of adolescent health 2006; 38:385-394.

[87] Thompson EA, Dahr J, Susan M Barron S. Setting standards in homeopathic practice--a pre-audit exploring motivation and expectation for patients attending the Bristol Homeopathic Hospital. Journal of the Faculty of Homeopathy 2007; 96(4):243-246.

[88] Gozum S, Arikan D, Buyukavci M. Complementary and Alternative Medicine Use in Pediatric Oncology Patients in Eastern Turkey. Cancer Nursing 2007; 30(1).

[89] Hanson E, Kalish LA, Bunce E, Curtis C, McDaniel S et al. Use of complementary and alternative medicine among children diagnosed with autism spectrum disorder. [References]. Journal of Autism and Developmental Disorders 2007; 37(4):628-636.

[90] Adams SK, Murdock KK, McQuaid EL. Complementary and Alternative Medication (CAM) Use and Asthma Outcomes in Children: An Urban Perspective. Journal of Asthma 2007; 44:775-782.

[91] Robinson N, Blair M, Lorenc A, Gully N, Fox P et al. Complementary medicine use in multi-ethnic paediatric outpatients. Complement Ther Clin Pract 2008 Feb;14(1):17-24 2008.

[92] Vlieger AM, Blink M, Tromp E, Benninga MA. Use of complementary and alternative medicine by pediatric patients with functional and organic gastrointestinal diseases: results from a multicenter survey. Pediatrics 2008; 122(2):e446-e451.

[93] Shaw A, Noble A, Salisbury C, Sharp D, Thompson E et al. Predictors of complementary therapy use among asthma patients: results of
a primary care survey. Health and Social Care in the Community 2008; 16(2):155-164.

[94] Bull L. Survey of complementary and alternative therapies used by children with specific learning difficulties (dyslexia). International Journal of Language & Communication Disorders 2009;44(2):224-35 2009.

[95] Post-White J, Fitzgerald M, Hageness S, Sencer SF. Complementary and alternative medicine use in children with cancer and general and specialty pediatrics. Journal of Pediatric Oncology Nursing 2009; 26(1):7-15.

[96] Tanase A, Zanni R. The use of complementary and alternative medicine among pediatric cystic fibrosis patients. J Altern Complement Med 2008 Dec;14(10):1271-3 2008.

[97] Davis MF, Meaney FJ, Duncan B. Factors influencing the use of complementary and alternative medicine in children. Journal of Alternative & Complementary Medicine - New York 2004 Oct;10(5):740-2 2004.

[98] Cincotta DR, Crawford NW, Lim A, Cranswick NE, Skull S et al. Comparison of complementary and alternative medicine use: reasons and motivations between two tertiary children's hospitals. Arch Dis Child 2006; 91(2):153-158.

[99] Yoon SL, Black S. Comprehensive, integrative management of pain for patients with sickle-cell disease. Journal of Alternative & Complementary Medicine - New York 2006 Dec;12(10):995-1001 2006.

[100] Maclennan AH, Myers SP, Taylor AW. The continuing use of complementary and alternative medicine in South Australia: costs and beliefs in 2004. Medical Journal of Australia 2006 Jan 2;184(1):27-31 2006.

[101] McCann LJ, Newell SJ. Survey of paediatric complementary and alternative medicine use in health and chronic illness. Archives of Disease in Childhood 2006 Feb;91(2):173-4 2006.

[102] He HG, Polkki T, Pietila AM, Vehvilainen-Julkunen K. Chinese parent's use of nonpharmacological methods in children's postoperative pain relief. Scandinavian Journal of Caring Sciences 2006 Mar;20(1):2-9 2006.

[103] Hurvitz EA, Leonard C, Ayyangar R, Simson N, V. Complementary and alternative medicine use in families of children with cerebral palsy. Developmental Medicine and Child Neurology 2003 Jun;45(6):364-70 2003.

[104] Hagen LE, Schneider R, Stephens D, Modrusan D, Feldman BM. Use of complementary and alternative medicine by pediatric rheumatology patients. Arthritis Care and Research 2003 Feb 15;49(1):3-6 2003.

[105] Sibinga EM, Shindell DL, Casella JF, Duggan AK, Wilson MH. Pediatric patients with sickle cell disease: use of complementary and alternative therapies. J Altern Complement Med 2006 Apr;12(3):291-8 2006.

[106] Genc RE, Senol S, Turgay AS, Kantar M. Complementary and alternative medicine used by pediatric patients with cancer in Western Turkey. Oncology Nursing Forum 2009; 36(3):284.

[107] Wong VC. Use of complementary and alternative medicine (CAM) in autism spectrum disorder (ASD): Comparison of Chinese and Western culture (Part A). Journal of Autism and Developmental Disorders 2009; 39(3):454-463.

[108] McCarty RL, Weber WJ, Loots B, Breuner CC, Vander Stoep A et al. Complementary and alternative medicine use and quality of life in pediatric diabetes. Journal of Alternative & Complementary Medicine 2010; 16(2):165-173.

[109] Carlton P, Johnson I, Cunliffe C. Factors influencing parents' decisions to choose chiropractic care for their children in the UK. Clinical Chiropractic 2009; 12(1):11-22.

[110] Zuzak TJ, Zuzak-Siegrist I, Rist L, Staubli G, Simoes-Wust AP. Medicinal systems of complementary and alternative medicine: a cross-sectional survey at a pediatric emergency department. journal 2010.
